# Evidence-Based Nutraceuticals in Human Health and Longevity Medicine

**DOI:** 10.3390/nu18060935

**Published:** 2026-03-16

**Authors:** Michele Antonelli, Davide Donelli

**Affiliations:** 1 Studio Medico Dott. Michele Antonelli, 42025 Cavriago, Italy; 2 Studio Cardiologico Dott. Davide Donelli, 42025 Cavriago, Italy; donelli.davide@gmail.com

According to the World Health Organization (WHO), all countries face major challenges in adapting health and social systems to the rapid aging of the population, as the proportion of people over 60 is expected to nearly double from 12% to 22% between 2015 and 2050, and since the year 2020 older adults have already outnumbered children under 5 years globally [1]. The rapid demographic shift toward an aging global population has intensified the need for developing effective strategies aimed not only at disease treatment, but at the preservation of functional capacity and quality of life across the lifespan.

Longevity medicine, an emerging, evidence-driven discipline focused on targeting the biological mechanisms of aging and disease prevention, has increasingly recognized nutrition as an important component of preventive and personalized care [2,3]. In fact, evidence from large cohort studies highlights that maintaining a healthy body weight across the course of life and prioritizing the quality and source of macronutrients over their absolute quantity are central determinants of longevity and healthy aging [4]. Dietary patterns rich in minimally processed plant foods, healthy fats, and limited red meats and added sugars (such as Mediterranean, Nordic, and Asian models) consistently show associations with reduced chronic disease risk and mortality while allowing cultural adaptability and personalization [5,6,7,8]. Greater consumption of polyphenol-rich foods (such as leafy greens, legumes, nuts, seeds, berries, olive oil, and green tea) may enhance cardiometabolic and cognitive health by modulating inflammatory and oxidative processes and shaping gut microbiota composition [9,10,11]. In parallel, adequate intake of essential micronutrients, including vitamins and minerals (through diet and, when necessary, targeted supplementation) supports genomic stability, mitochondrial function, antioxidant defenses, and epigenetic regulation [12]. Diets rich in fiber and plant bioactives promote microbial diversity and metabolic homeostasis, while overall caloric balance and dietary quality influence key nutrient-sensing pathways implicated in aging (such as AMPK, sirtuins, and mTOR) thereby fostering autophagy and cellular resilience [13].

In particular, emerging evidence identifies gut dysbiosis as a key feature of aging, characterized by a decline in beneficial microbial metabolites such as short-chain fatty acids and indole derivatives that support metabolic health, alongside an increase in pathobiont-derived metabolites linked to insulin resistance and atherosclerosis [14]. These microbiota changes are more pronounced in unhealthy aging, whereas longevity is associated with microbial signatures enriched in beneficial metabolite producers [15]. Diet plays a central role in shaping these pathways, with plant-based foods supporting microbial diversity, while microbiota-targeted strategies—including supplementation with prebiotics, probiotics, and postbiotics when needed—may represent promising adjunctive approaches to promote metabolic health and healthy aging [16,17,18].

From a public health perspective, creating supportive food environments, reducing ultra-processed food consumption, integrating environmental sustainability into dietary guidance, and advancing research in diverse populations with improved biomarkers are essential to align individual longevity strategies with broader societal and planetary health goals [19,20,21,22]. Moreover, ensuring equitable access to healthy foods and integrating personalized nutrition into clinical practice can reduce the socioeconomic burden of population aging [23].

Within this framework, nutraceuticals, used as complementary tools to nutritional and lifestyle interventions, and which include vitamins, minerals, amino acids, functional foods, probiotics, plant-derived bioactive compounds, and other substances (see Table 1 for further details), represent a promising interface between molecular science, health promotion, and integrative medicine [24,25]. Importantly, nutraceuticals should not be viewed as substitutes for healthy lifestyle behaviors, but rather as adjunctive tools that can support the effects of established interventions such as balanced nutrition, regular physical activity, adequate sleep, stress management, and avoidance of harmful exposures [26]. Lifestyle modification remains the cornerstone of longevity-oriented medicine and chronic disease prevention; however, there are real-world contexts (such as individual predisposition, environmental constraints, or reduced adherence capacity) in which lifestyle measures alone may be insufficient, impractical, or only partially effective. In such settings, evidence-based nutraceuticals may provide targeted support by addressing specific nutritional deficiencies, modulating biological pathways potentially implicated in disease development, or complementing conventional preventive and therapeutic strategies [27].

This Special Issue of *Nutrients*, entitled “Nutraceuticals’ Role in Promoting Human Health and Well-Being: An Evidence-Based Perspective”, was developed to critically examine the role of nutraceuticals through the lens of scientific rigor. A recurring theme across the collected articles is the importance of moving beyond anecdotal use toward evidence-based applications of nutraceuticals.

The original research articles of the Special Issue illustrate diverse and innovative approaches to evidence-based nutraceutical research. Baldassano et al. investigated the biofortification of vegetables with iodine and molybdenum, highlighting a sustainable nutritional strategy to prevent micronutrient deficiencies with potential long-term implications for metabolic health [33]. Campos et al. explored lifestyle and dietary patterns among food supplement users in the Portuguese population, providing valuable insights into real-world supplement use and its intersection with health behaviors [34]. Moreover, Kim and Baik examined the effects of a plant-derived nutraceutical—specifically hawthorn fruit supplementation—on facial skin aging phenotypes and leukocyte telomere length, stratified by genetic polymorphisms, thereby contributing to the growing field of nutrigenomics and personalized longevity interventions [35].

The review articles further expand the clinical and mechanistic scope of the Special Issue. Ribeiro et al. provided a focused scoping review on nutritional approaches in neurodegenerative disorders, with particular emphasis on Spastic Paraplegia 11 (SPG11), linking metabolic dysregulation and neurodegeneration [36]. Markowska et al. reviewed the potential role of epigallocatechin gallate, a well-characterized plant polyphenol, in endometriosis, underscoring how nutraceuticals may modulate inflammatory and proliferative pathways in chronic conditions [37]. Finally, Antonelli et al. conducted a systematic review on quail egg-based supplements in allergic rhinitis, exemplifying the application of evidence synthesis to evaluate integrative remedies within clinical practice [38].

Collectively, the contributions to this Special Issue reinforce the concept that nutraceuticals can play a meaningful role in integrative and longevity-oriented medicine when grounded in robust scientific evidence. Rather than serving as stand-alone solutions, these interventions are best positioned as components of integrated, personalized strategies that combine lifestyle modification and conventional medical care to promote healthy aging and reduce the burden of chronic diseases.

We hope that this Special Issue will stimulate further high-quality research, support informed clinical decision-making, and contribute to the responsible development of nutraceuticals within the evolving field of longevity medicine.

## Figures and Tables

**Table 1:** Main types of nutraceuticals and dietary supplements [28,29,30,31,32].

Functions	Examples	Type
Support immune, bone, neurological, and antioxidant functions	Vitamin C, Vitamin D, Vitamin B-complex, Vitamin E	Vitamins
Support bone health, enzymatic activity, immune function, and oxygen transport	Calcium, Magnesium, Zinc, Selenium, Iron	Minerals
Reduce inflammation, support cardiovascular and cognitive health	Omega-3 fatty acids (EPA, DHA), fish oil, algal oil	Polyunsaturated fatty acids and marine-derived lipids
Support muscle protein synthesis, physical performance, nitric oxide production, and metabolic health	Leucine, arginine, creatine, whey and plant proteins	Amino acids and protein-related supplements
Support gut microbiota balance, intestinal barrier function, immune and metabolic regulation	Inulin, *Lactobacillus*, *Bifidobacterium*, butyrate	Prebiotics, probiotics, and postbiotics
Provide health benefits beyond basic nutrition and support cardiometabolic health	Yogurt with probiotics, fortified foods, oats, plant sterol-enriched foods	Functional foods
Provide antioxidant, anti-inflammatory, immunomodulatory, and metabolic regulatory effects	Green tea polyphenols, curcumin, resveratrol, quercetin, mushroom-derived beta-glucans	Plant- and mushroom-derived bioactive compounds
Support immune modulation and tissue repair functions	Royal jelly, colostrum, egg-derived phospholipids	Animal-derived products
Support mitochondrial function, cardiovascular health, circadian rhythm regulation, and cellular aging-related pathways	Coenzyme Q10, melatonin	Other bioactive and longevity-related compounds

## Data Availability

All data are available by contacting the corresponding author.
